# Characterization of two related *Erwinia* myoviruses that are distant relatives of the PhiKZ-like Jumbo phages

**DOI:** 10.1371/journal.pone.0200202

**Published:** 2018-07-06

**Authors:** Daniel K. Arens, T. Scott Brady, John L. Carter, Jenny A. Pape, David M. Robinson, Kerri A. Russell, Lyndsay A. Staley, Jason M. Stettler, Olivia B. Tateoka, Michelle H. Townsend, Kiara V. Whitley, Trevor M. Wienclaw, Taryn L. Williamson, Steven M. Johnson, Julianne H. Grose

**Affiliations:** 1 Microbiology and Molecular Biology Department, Brigham Young University, Provo, Utah, United States of America; 2 Plant and Wildlife Sciences Department, Brigham Young University, Provo, Utah, United States of America; Niels Bohr Institute, DENMARK

## Abstract

Bacteriophages are a major force in the evolution of bacteria due to their sheer abundance as well as their ability to infect and kill their hosts and to transfer genetic material. Bacteriophages that infect the *Enterobacteriaceae* family are of particular interest because this bacterial family contains dangerous animal and plant pathogens. Herein we report the isolation and characterization of two jumbo myovirus *Erwinia* phages, RisingSun and Joad, collected from apple trees. These two genomes are nearly identical with Joad harboring two additional putative gene products. Despite mass spectrometry data that support the putative annotation, 43% of their gene products have no significant BLASTP hit. These phages are also more closely related to *Pseudomonas* and *Vibrio* phages than to published *Enterobacteriaceae* phages. Of the 140 gene products with a BLASTP hit, 81% and 63% of the closest hits correspond to gene products from *Pseudomonas* and *Vibrio* phages, respectively. This relatedness may reflect their ecological niche, rather than the evolutionary history of their host. Despite the presence of over 800 *Enterobacteriaceae* phages on NCBI, the uniqueness of these two phages highlights the diversity of *Enterobacteriaceae* phages still to be discovered.

## Introduction

The existence of bacteriophages has been known since the early 1900’s when Frederick Twort and Felix d’Herelle independently isolated phage [1]. Phages are now considered the most abundant source of biomass on the planet [2] and contribute heavily to the evolution of bacteria because of their ability to infect and lyse different bacterial strains as well as their ability to transfer genetic information [3, 4]. Due to this transfer of genetic information, phages have been shown to be required for the pathogenicity of several bacterial strains, such as pathogenic *V*. *cholerae*, *C*. *diphtheriae*, and *E*. *coli* strains [5–7]. This incredible bacterial host specificity has led to many attempts to use them as diagnostic and therapeutic agents [8]. d’Herelle was one of the first to put this into practice by using phage to treat and cure patients with *Bacillus* based dysentery [9].

*Enterobacteriaceae* is one of the most highly studied bacterial families with over 50 accepted genera. These genera include several well-known animal pathogens such as *Enterobacter*, *Escherichia*, *Klebsiella*, *Salmonella*, *Shigella*, and *Yersinia*, as well as plant pathogens such as *Erwinia* and *Dickeya (for a recent discussion on the classification of this family see [10])*. This family is a major health concern in the United States with the Centers for Disease Control and Prevention (CDC) citing carbapenem resistant and extended spectrum β-lactamase (ESBL) producing *Enterobacteriaceae* as “urgent” and “serious” threats [11]. Plant pathogens are also of great concern, with *Erwinia* infections causing over $100 million in agricultural loss per year [12]. Studying the evolution of this family of bacteria, including the phages that infect them, is critical to controlling many health and agricultural concerns.

Over 800 phages that infect members of the *Enterobacteriaceae* family have been isolated, sequenced, and deposited in NCBI. Most of these phages infect genera with common animal pathogens including *Escherichia*, *Salmonella*, *Shigella*, and *Klebsiella*. Phages of the plant pathogens have also been recently deposited, including those that infect *Erwinia amylovora*, the causative agent of fire blight [13]. When infected, fire Blight causes the leaves of the *Rosaceae* plants to dry and shrivel up, giving the appearance of being scorched. Another similar wilting disease infecting the *Cucurbita* genus, which includes squash and pumpkin, is caused by *Erwinia tracheiphila*. It has been reported to cause millions of dollars of agricultural loss in the northeastern United States [14]. Therefore, the study of *Erwinia* phages may aid in understanding and treating multiple devastating agricultural diseases. Currently, 45 *Erwinia* specific phages have been isolated and deposited on NCBI, 25 of which were discovered by our group.

Herein we report the isolation and characterization of two of these *Erwinia* phages, vB_EamM_RisingSun (RisingSun) and vB_EamM_Joad (Joad). These phages have only very distant relationships to other published phages and are highly similar to one another, with Joad containing two additional genes. BLASTP hits to putative annotated ORFs indicate that much of their genomes are composed of novel proteins with no BLASTP hit, while many of those having BLASTP hits harbor closer relationships to *Pseudomonas* and *Vibrio* phages as opposed to *Enterobacteriaceae* phages.

## Methods

### Phage isolation, sequencing and host range

Both Joad and RisingSun were isolated from apple blossom samples collected in Payson, Utah. Blossoms were crushed with a mortar and pestle and the resulting debris was added to an exponential culture of *Erwinia amylovora* ATCC 29780 [15, 16]. The enrichment culture was harvested by centrifugation 48 hours later, filtered with at 0.45 μM filter, and used to infect a fresh culture of bacteria. Three plaque purifications were performed, after which a high titer lysate was made in liquid broth. Phage DNA was extracted from this lysate using the Phage DNA Isolation Kit (Norgen Biotek Corporation), and was sequenced, assembled, and annotated as previously described [15]. Host range was performed by spotting 5 uL of lysate onto 0.5 mL of bacteria imbedded in LB top agar. Positive spots were verified by plaque assay. Bacterial strains used included *Erwinia amylovora* ATCC 29780, *Erwinia amylovora* EA110 [16], *Pantoea agglomerans* E325 [17], *Pantoea vegans* C9-1 [18], *Dickeya chrysanthemi* ATCC 11663 [19], the common clinical strain *Pseudomonas aeruginosa* Boston 41501 ATCC 27853 [20], *Pseudomonas chlororaphis* ATCC13985 [21], *Vibrio cholerae* ATCC 14035 (originally deposited by Standards Lab., London) [22], *Salmonella enterica typhimurium* LT2 (a generous gift from John Roth, UCDavis), *Enterobacter cloacae* ATCC 13047(deposited by the CDC) [23], and *E*. *coli* BW25113 [24].

### Electron microscopy

Samples were prepared for transmission electron microscopy by placing 20 uL of high-titer phage lysate on a 200-mesh copper carbon type-B electron microscope grid for two to five minutes. Excess lysate was wicked away and the grid was then stained for one minute using 2% phosphotungstic acid. The grid was then briefly dipped into distilled water and excess liquid was wicked away. Phages were imaged at the BYU Microscopy Center. The phages were measured for capsid width as well as tail length and width using ImageJ software [25].

### Mass spectrometry

Samples were prepared according to the methods of Guttman et al. [26]. Briefly, (all concentrations are final concentrations) fresh lysates were diluted with TNE (50 mM Tris pH 8.0, 100 mM NaCl, 1 mM EDTA) buffer and RapiGest SF reagent (Waters Corp.) was added to 0.1% before 5 min of boiling. Next, samples were incubated at 37°C for 30 min in the presence of 1 mM TCEP (Tris (2-carboxyethyl) phosphine). Iodoacetamide (0.5 mg/ml) was used to carboxymethylate the samples for 30 min at 37^0^ C. Carboxymethylation was neutralized with 2 mM TCEP. Using a trypsin:protein ratio of 1:50 samples were digested overnight at 37^0^ C. Next, 250 mM HCl was used to degrade the RapiGest for 1 hr at 37^0^ C. Samples were then centrifuged for 30 min at 4^0^ C and 14000 rpm. In new tubes, peptides were extracted from the soluble fractions by desalting using Aspire RP30 desalting columns (Thermo Scientific).

High pressure liquid chromatography (HPLC) coupled with tandem mass spectroscopy (LC-MS/MS) using nano-spray ionization was used to analyze the trypsin-digested peptides according to the method of McCormack et al. [27]. Experiments were performed on a TripleTOF 5600 hybrid mass spectrometer (ABSCIEX) interfaced with nano-scale reversed-phase HPLC (Tempo) using a 10 cm-100 μm ID glass capillary packed with 5-μm C18 Zorbax^TM^ beads (Agilent Technologies, Santa Clara, CA). The peptides were eluted from the C18 packed capillary tubes into the mass spectrometer using a linear gradient of Acetonitrile (ACN) (5–60% generated from two buffers: buffer A with 98% H_2_O, 2% ACN, 0.2% formic acid, and 0.005% TFA, and buffer B with 100% ACN, 0.2% formic acid, and 0.005% TFA) at a flow rate of 250 μl/min for 1 hr.

MS/MS data were acquired in a data-dependent manner in which the MS1 data were acquired for 250 ms at m/z of 400 to 1250 Da and the MS/MS data were acquired from m/z of 50 to 2,000 Da. For independent data acquisition (IDA) parameters of MS1-TOF for 250 milliseconds, followed by 50 MS2 events of 25 milliseconds each were used. The IDA criteria were set at over 200 counts threshold, charge state of plus 2–4 with 4 seconds exclusion window. Finally, the collected data were analyzed using MASCOT^®^ (Matrix Sciences) and Protein Pilot 4.0 (ABSCIEX) for peptide identifications.

### Analysis of RisingSun and Joad phage genomes

Gepard [28] was used to create three dot plots, one with whole genome sequences, one with major capsid protein (MCP) sequences, and one with terminase sequences. Putative major capsid proteins and terminase proteins from phage Joad were used in BLASTP [29–31] analysis to find related phages [2, 32, 33]. Accession numbers that were used are: whole genome accession numbers (Joad [MF459647], RisingSun [MF459646], Pseudomonas phage EL [NC_007623.1], Pseudomonas phage OBP [NC_016571.1], Vibrio phage pTD1 [AP017972.1], Vibrio phage VP4B [KC131130.1], Pseudomonas phage phiKZ [AF399011.1]), MCP accession numbers (Joad [ASU03832.1], RisingSun [ASU03587.1], *Pseudomonas* phage EL [YP_418111.1], *Vibrio* phage pTD1 [BAW98274.1], *Vibrio* phage VP4B [AGB07257.1], *Pseudomonas* phage OBP [YP_004958031.1], *Pseudomonas* phage phiKZ [AAL83021.1]) and terminase accession numbers (Joad [ASU03673.1], RisingSun [ASU03430.1] *Pseudomonas* phage EL [YP_418044.1], *Vibrio* phage pTD1 [BAW98365.1], *Vibrio* phage VP4B [AGB07167.1],*Pseudomonas* phage OBP [YP_004957913.1], *Pseudomonas* phage phiKZ [NP_803591.1]). Kalign [34–38] was used to determine average nucleotide identity (ANI) of the phage genomes.

### Motif analysis and identification

The Center for Phage Technology Galaxy Server (https://cpt.tamu.edu/galaxy-pub/) and MEME [39] were used to scan the phage genome for significant motifs with an e-value less than 10^−7^. The Galaxy Server was able to scan the entire genome at once. FIMO [40] was used to search the phage genome for motifs found in the Galaxy results that passed our significance threshold (q value <0.01) and determined the exact positions of the motif(s) in the entire genome. We then used DNA Master [41] and Phamerator [42] to analyze the genes neighboring the motifs to determine putative transcription patterns.

## Results and discussion

### Phage isolation and sequencing

Joad and RisingSun were isolated from apple tree samples that appeared to be infected with fire blight. DNA analysis suggests Joad and RisingSun are Jumbo phages [43] with genome sizes of 235374 bp and 235108 bp respectively (a summary of their genomes is provided in Table 1). A search for tRNA’s using tRNA ScanSE [44] returned no tRNA results. No rigorous testing for lysogeny formation has been performed, but their clear plaque morphology and ease in obtaining higher titers suggest they may be lytic phages. This conclusion is supported by a BLASTP analysis of the putative Major Capsid Protein (MCP) from Joad, which has only close BLASTP hits to phage and none to bacterial genomes. In a recent analysis of phage, Casjens et al. found that MCP’s from temperate phages generally have BLASTP hits that are >70% identity in bacterial genomes [45].

**Table 1:** General characteristics of *Erwinia amylovora* phages Joad and RisingSun.

Phage Name	GenBank Accession #	Fold Coverage	Genome Length (bps)	ORFs	tRNAs	GC%
Joad	MF459647	522.2	235374	245	none	48.29
RisingSun	MF459646	138.6	235108	243	none	48.32

### Phage morphology

**Fig 1:**
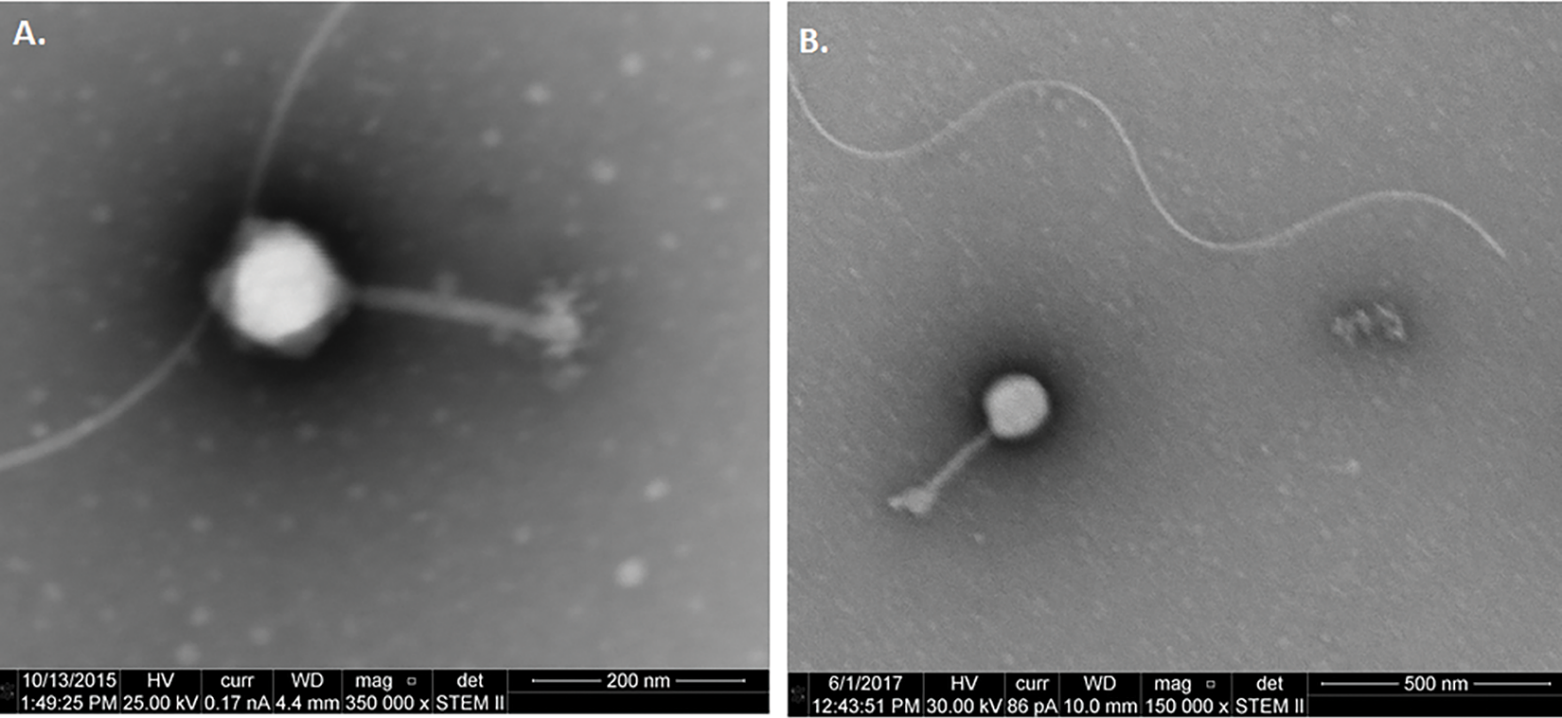
Transmission electron microscopy revealed (A) RisingSun and (B) Joad as *Myoviridae*.

Transmission electron microscopy (TEM) analysis revealed the large nature of these phages. From three independent TEM images of RisingSun (Fig 1), the average capsid width was 143.2 +/- 6.0 nm, tail width was 23.2 +/- 2.4 nm, and tail length was 206.8 +-3.6 nm, consistent with the large genome size reported above. The measurements from the single Joad image obtained were within the standard deviation of RisingSun measurements. The viral morphology, icosahedral capsids attached to long contractile tails, is consistent with these phages belonging to the jumbo *myoviridae* bacteriophages [32].

### Genomic analysis

**Fig 2:**
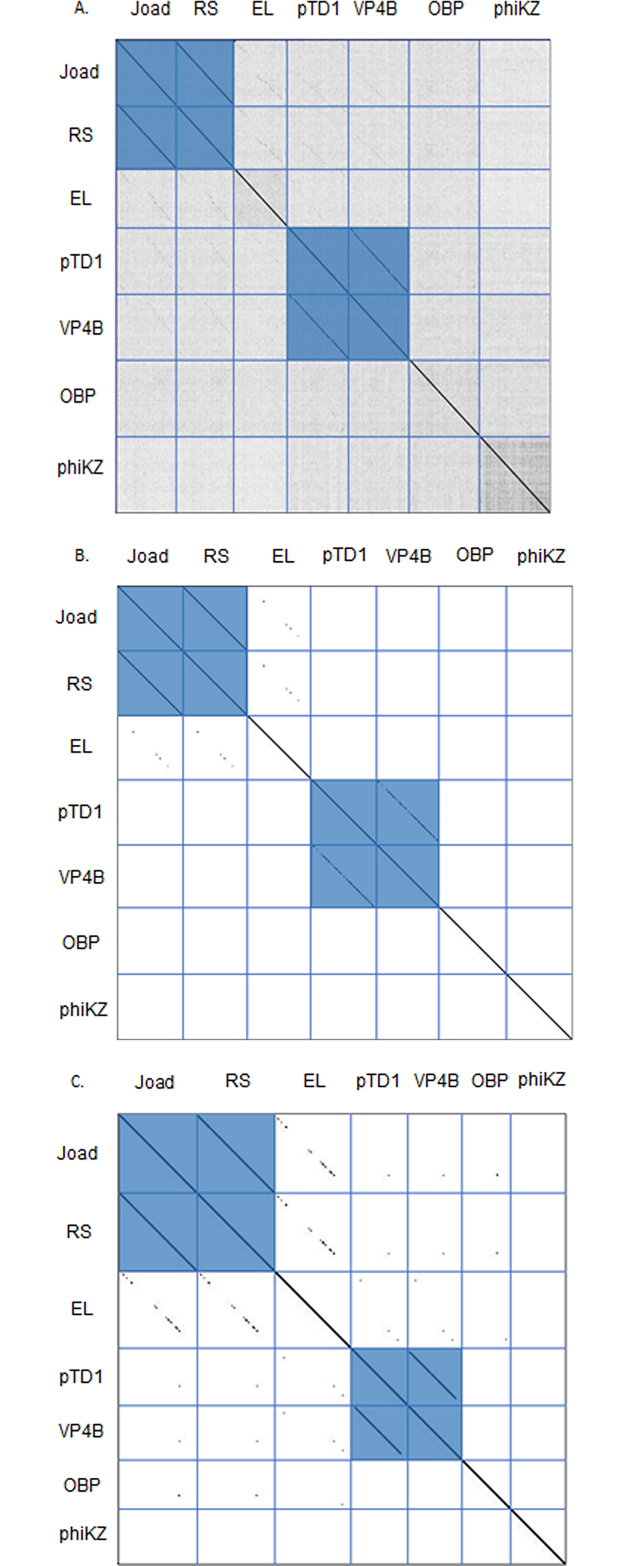
Dot plot comparisons for whole genome nucleotide sequences, MCP amino acid sequences, and terminase amino acid sequences of seven phages. Blue horizontal and vertical lines were added to show genome boundaries. *Erwinia* phages: Joad, RisingSun (RS). *Vibrio* phages: pTD1, VP4B. *Pseudomonas* phages: EL, OBP, phiKZ. **A)** Nucleotide dot plot shows genome similarity between seven phage genomes. Two distinct clusters are shown, the Joad and RS cluster and the Vibrio phage cluster. **B)** Dot plot comparison for MCP amino acid sequences of seven phages. The clusters are shown between Joad and RS and between *Vibrio* phages pTD1 and VP4B. **C)** Dot plot comparison for terminase amino acid sequences of seven phages. Two clusters are formed between Joad and RS and between *Vibrio* phages pTD1 and VP4B.

Whole genome dot plot comparisons (Fig 2A) were performed using the whole genome sequences of Joad, RisingSun, and any phages that were retrieved from a BLASTP analysis of their putative major capsid (MCP) and terminase proteins. The dot plot reveals two distinct clusters of phages, and low relatedness between singletons (unique phages unrelated to others in the group). Clusters are defined here similarly to other studies, as two or more phages with sequence similarity over at least half of the genome [2]. Joad and RisingSun constitute one cluster with *Pseudomonas* phage EL being a distant relation in the gray area of cluster boundary, while the second cluster is comprised of two *Vibrio* phages, VP4B and pTD1. Whole genome Average Nucleotide Identity (ANI) data shown in Table 2A support the clusters identified in Fig 2A with a 96% identity match between Joad and RisingSun. These two phages also had 46% similarity compared to EL and only 36% similarity compared to OBP and phiKZ, while low similarity is seen between Joad and the singleton phages. A 75% identity match is also observed within the vibrio phage cluster of VP4B and pTD1. ANI data show that Joad and RisingSun differ in genome size by 266 nucleotides, Joad having the larger genome, in an otherwise highly similar genome (~96.6% nucleotide identity). In addition to the distinct clusters, Fig 2A shows a distant relationship between Joad and EL, indicative of divergent evolution and lateral gene transfer that has occurred.

**Table 2:** Joad and RisingSun are a unique cluster of phages when compared to others using whole genomes, MCP amino acid sequences, and terminase amino acid sequences. **2A)** Similarity of seven phage genomes according to Average Nucleotide Identity (ANI). **2B)** Percent identity of major capsid proteins (MCP) amongst seven phages. **2C)** Percent identity of terminase proteins amongst seven phages. Percent identity was determined by BLASTP analysis.

**2A**	**ANI of Whole genome**						
	**Joad**	**Rising Sun**	**EL**	**pTD1**	**VP4B**	**OBP**	**phiKZ**
**Joad**	100						
**RisingSun**	96.61	100					
**EL**	45.83	45.73	100				
**pTD1**	38.24	37.86	35.21	100			
**VP4B**	38.8	38.41	35.67	75.14	100		
**OBP**	36.53	36.17	33.7	37.08	41.11	100	
**phiKZ**	36.25	35.94	33.5	37.04	41.2	46.98	100
**2B**	**Identity of MCP gene products**						
	**Joad**	**Rising Sun**	**EL**	**pTD1**	**VP4B**	**OBP**	**phiKZ**
**Joad**	100						
**RisingSun**	100	100					
**EL**	57.22	57.22	100				
**pTD1**	40.78	40.78	21.98	100			
**VP4B**	40.48	40.48	38.27	89.74	100		
**OBP**	30.71	30.71	31.18	33.61	34.90	100	
**phiKZ**	23.58	23.58	21.98	21.60	22.19	25.29	100
**2C**	**Terminase gene products**						
	**Joad**	**Rising Sun**	**EL**	**pTD1**	**VP4B**	**OBP**	**phiKZ**
**Joad**	100						
**RisingSun**	99.63	100					
**EL**	56.43	56.43	100				
**pTD1**	53.63	53.63	50.42	100			
**VP4B**	53.96	53.96	50.08	95.75	100		
**OBP**	48.49	48.49	48.31	47.59	47.97	100	
**phiKZ**	27.71	27.71	27.29	31.54	32.24	28.73	100

A dot plot of the Major Capsid Protein (MCP) and terminase amino acid sequences (Fig 2B & 2C) and percent identity data (Table 2B) support this distant relationship between Joad and EL. Table 2B shows a 57% identity between the MCP amino acid sequences of the two phages, whereas *Vibrio* phages pTD1 and VP4B MCP amino acid sequences are highly similar at nearly 90% amino acid identity. The terminase dot plot, Fig 2C, and percent identity data in Table 2C continue to support a distant relationship between Joad and EL.

Both the dot plot and ANI indicate that the Joad cluster is markedly different from the *Vibrio* cluster and EL phage consistent with the weak similarity of the MCP and terminase amino sequences. Comparing Joad to OBP and phiKZ, which have similar terminase proteins, shows little relation and confirms the distinctiveness of Joad. Since terminase conservation has been shown to reflect phage packaging mechanisms [46] the terminase similarity to phiKZ, although distant (~28% identity) suggests these phages package DNA by a headful mechanism [47]. This analysis is consistent with analysis of our whole-genome phage sequencing raw results for RisingSun analyzed by Phageterm [48] which also suggested a headful packaging mechanism.

### Whole-proteome comparison of Joad and RisingSun

**Fig 3:**
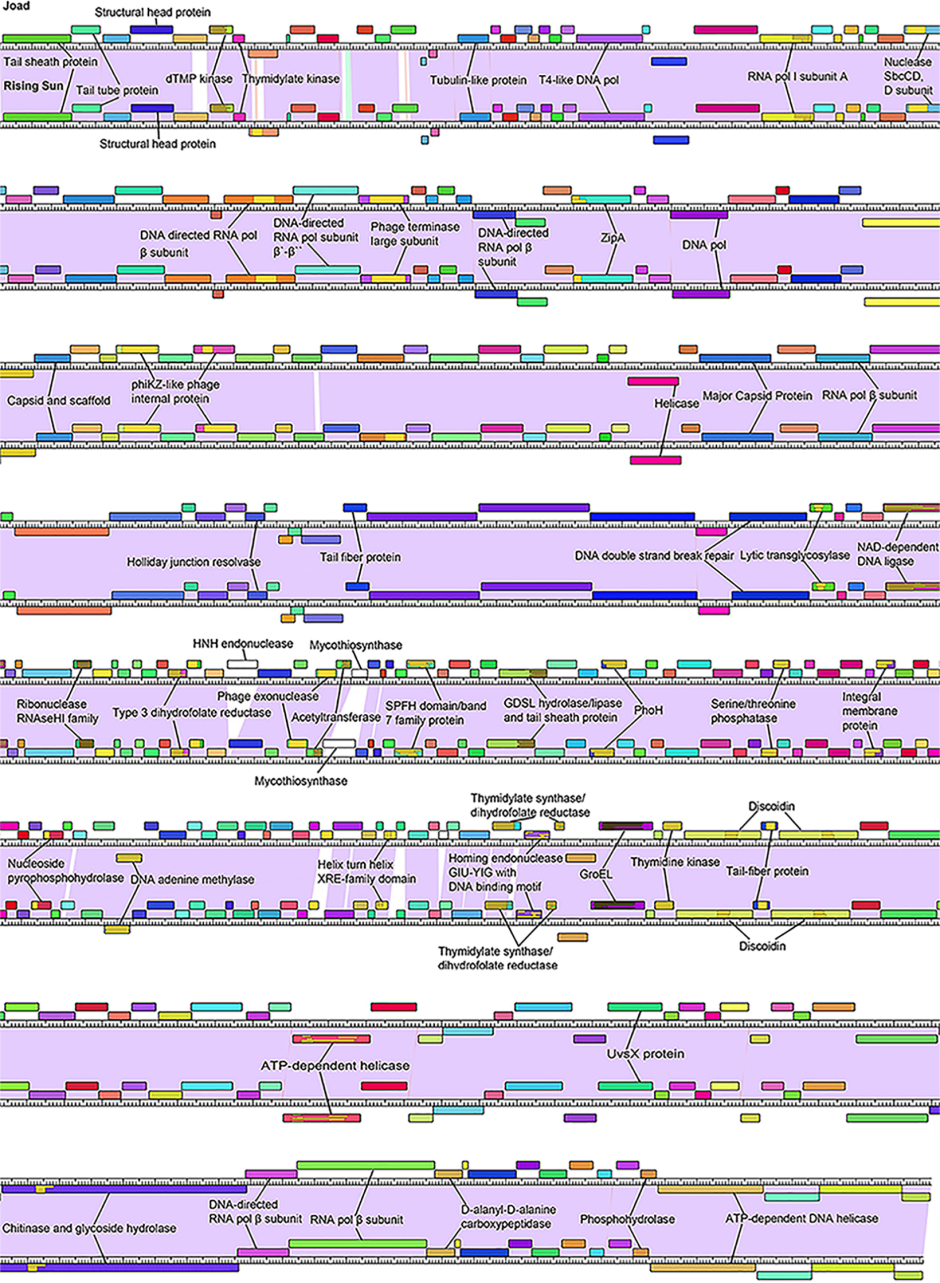
Whole genome comparison map between the phages Joad and RisingSun adapted from Phamerator [42]. Boxes on top of the genome ruler are genes expressed on the forward strand, while boxes under the genome ruler are genes expressed on the reverse strand. The colored boxes categorize homologous proteins. The purple between the two genomes represents high nucleotide similarity, while the white between the two nucleotides represents nucleotide variation. Annotated functions were collected through BLAST and Phamerator searches. Abbreviations include: Discoidin (Coagulation factor also known as F5/8 type C domain known as the discoidin (DS) domain family); GroEL (GroEL-like type 1 chaperonin protein); PhoH (Phosphate starvation protein PhoH); ZipA (cell division protein ZipA).

Phamerator [42] was used to produce full-genome comparison maps for both Joad and RisingSun which were modified for simplicity (Fig 3). Consistent with the mild differences seen in ANI analysis, the genomes encode nearly identical gene products (colored with similar coloring based on the Phamerator default values of greater than 32.5% identity by BLASTP and less than 1e-50 e-value from ClustalO), with many having 100% identity. The most obvious difference is the two genes present in Joad that are not present in RisingSun, of which gene product 122 had a BLASTP hit to an HNH endonuclease and the other encodes a protein with unknown function. Not every gene with a BLASTP or Phamerator hit is labeled on this map as many have an unknown function or are indiscriminant structural proteins. Proteins of significance are discussed in the following section “Interesting proteins”, for which Fig 3 will serve as a reference.

### Interesting proteins and host range

**Fig 4:**
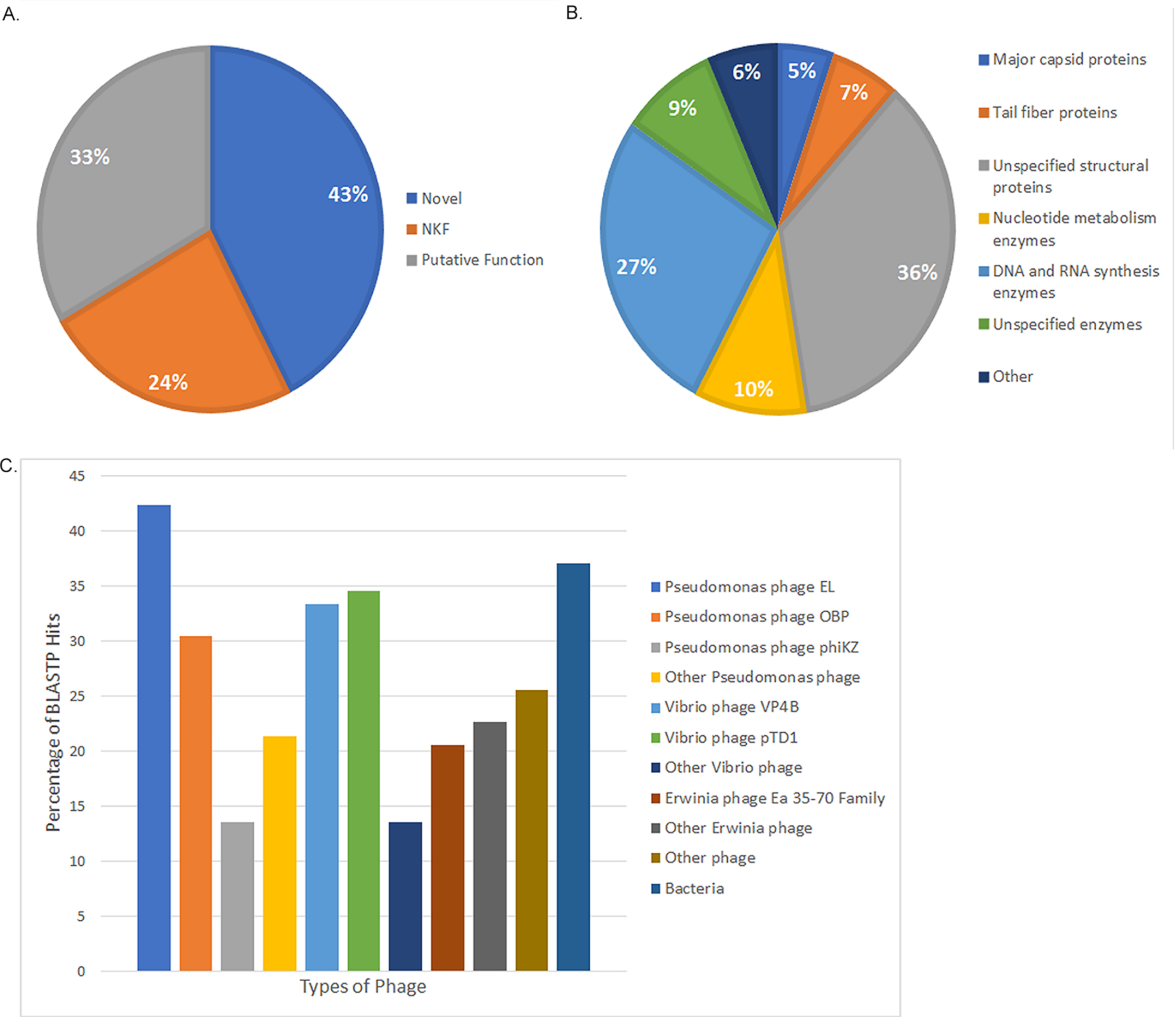
RisingSun and Joad are unique phages whose proteomes contain novel proteins. **A)** Distribution of proteins in RisingSun based upon BLASTP hits that are novel, have no known function, and putative function. **B)** Putative gene ontology in RisingSun. **C)** Percentage of RisingSun gene products with BLASTP hits to proteins in other phages/organisms.

Joad and RisingSun appear to be two very similar Jumbo phages [43] with only distant relationship to other phages. Due to the fact that these phages are so similar, we will be specifically referring to the genes, gene functions, and gene products of RisingSun. Of the 243 putative gene products in the RisingSun genome ~43% have no known function and do not have any significant BLASTP hits (e-value of 10^−7^ or less). This large proportion of proteins with no BLASTP hit (~43%) represents proteins that have not been previously found in nature [49, 50]. This finding, combined with their nucleotide dissimilarity discussed above, further sets RisingSun and Joad apart. The remaining gene products are represented as those with BLASTP hits but no known function (NKF) (~24%) and those with BLASTP hits and putative or known functions (~33%) (Fig 4A). Of the ~33% of gene products with known functions, ~36% of them are unspecified structural proteins and another ~12% represent major capsid and tail fiber proteins. The remaining gene products with putative function are primarily putative enzymes, namely those involved with DNA and RNA synthesis including an NAD-dependent DNA ligase (gp108), RNA polymerase beta subunit (gp29), and a helix-turn-helix XRE-family domain among others (gp180) (Fig 4B).

The RisingSun gene products that have BLASTP hits are mainly homologous to other phage gene products. Specifically, of the 140 gene products with BLASTP hits, 81% correspond to *Pseudomonas* phage gene products. Gene products from phages EL, OBP, and phiKZ match 74%, 53%, and 24% of NKF/putative function gene products, respectively, while other *Pseudomonas* phages match 37%. *Vibrio* phages also showed a marked similarity to the RisingSun proteome with 63% of gene products with a BLASTP hit from a *Vibrio* phage gene product. Phages VP4B and pTD1 match 58% and 60%, respectively, and other *Vibrio* phages match 24% of NKF/putative function gene products. Due to the high similarity of gene products with a BLASTP hit, we further analyzed the entire proteome of RisingSun in comparison to the *Pseudomonas* and *Vibrio* phages EL, OBP, VP4B and pTD1.

Despite the lack of strong nucleotide similarity (see Table 2), an analysis of total RisingSun gene homologs reveals 42% of RisingSun genes have homologs in *Pseudomonas* phage EL, 30% have homologs in *Pseudomonas* phage OBP, 33% in Vibrio phage VP4B and 35% in *Vibrio* phage pTD1 (see S1 Table for specific gene product homologs). In contrast RisingSun has only 14% of its gene products in common with phiKZ (Fig 4C). Note that these numbers are based off of annotation of gene products and could be different based on annotation. Given that other phage classifications systems have grouped related phage by 40% or greater proteome conservation, *Pseudomonas* phages EL and OBP as well as VP4B and pTD1 are distant members of a more evolutionarily diverse supercluster, with EL being the closest member to the Joad Cluster. The conserved gene products of this supercluster (totaling 63 gene products) are primarily structural genes (24 gene products) and include the MCP, portal, terminase and tail proteins. Nineteen of the remaining conserved gene products have putative functions in DNA replication and recombination, one appears to be involved in cell lysis (a phage related lysozyme). This leaves 19 of the 63 conserved gene products (~30%) that have no putative function. Due to this relationship, we tested the ability of phage Joad to infect *Pseudomonas aeruginosa* as well as several *Enterobacteriaceae* strains (Table 3). Although clear spots could be seen on *Erwinia amylovora*, *Pantoea vegans* and *Pseudomonas aeruginosa* Boston 41501 by spot test, no plaques were observed on *Pseudomonas aeruginosa* when assayed by plaque assay suggesting the spot test resulted from a toxin product in the Joad lysate. Joad did not appear to infect several other *Enterobacteriaceae* tested (including a *Pantoea agglomerans* strain, a *Vibrio cholerae* strain, an *E*. *coli* strain, a *Dickeya* strain, a *Salmonella* strain and an *Enterobacter* strain). As noted by the infectivity *Pantoea vegans* but not *Pantoea agglomerans* strain, this host range is no wise comprehensive since several other species or even strains within a species may be a host of Joad.

**Table 3:** Host range of phage Joad suggests narrow specificity towards *Erwinia amylovora* and the closely related *Pantoea vegans* bacteria. The bacteria tested is provided along with the plaque forming units from three independent assays.

Bacteria tested	Plaque forming units/mL (Pfu/mL)
*Erwinia amylovora* ATCC 29780	10^8^
*Erwinia amylovora* EA110	10^8^
*Pantoea vegans C9-1*	10^8^
*Pantoea agglomerans E325*	0
*Dickeya chrysanthemi* ATCC 11663	0
*Salmonella enterica serovar typhimurium* LT2	0
*E*. *coli* BW25113	0
*Enterobacter cloacae* ATCC 13047	0
*Pseudomonas aeruginosa* Boston 41501	0
*Pseudomonas chlororaphis* ATCC13985	0
*Vibrio cholerae ATCC 14035*	0

The RisingSun proteome also showed similarity to a range of other phage and bacteria. The most prominent group being a highly related *Erwinia* phage family, which consists of phages Deimos-Minion, Simmy50, SpecialG, and Ea35-70. This family matched to 21% of all RisingSun gene products, while gene products from other *Erwinia* phages corresponded to a total of 23% of gene products. Other phages infecting *Cronobacter* and *Ralstonia* bacteria among others matched 26% of the gene products, while 37% of total RisingSun gene products had a BLASTP hit to various bacteria including *Pseudomonas aeruginosa* (Fig 4C). While several of these less common matches had the lowest e-value from a particular BLAST, in a handful of cases they were for the most part orders of magnitude higher than the *Pseudomonas* and *Vibrio* phages discussed above.

**Fig 5:**
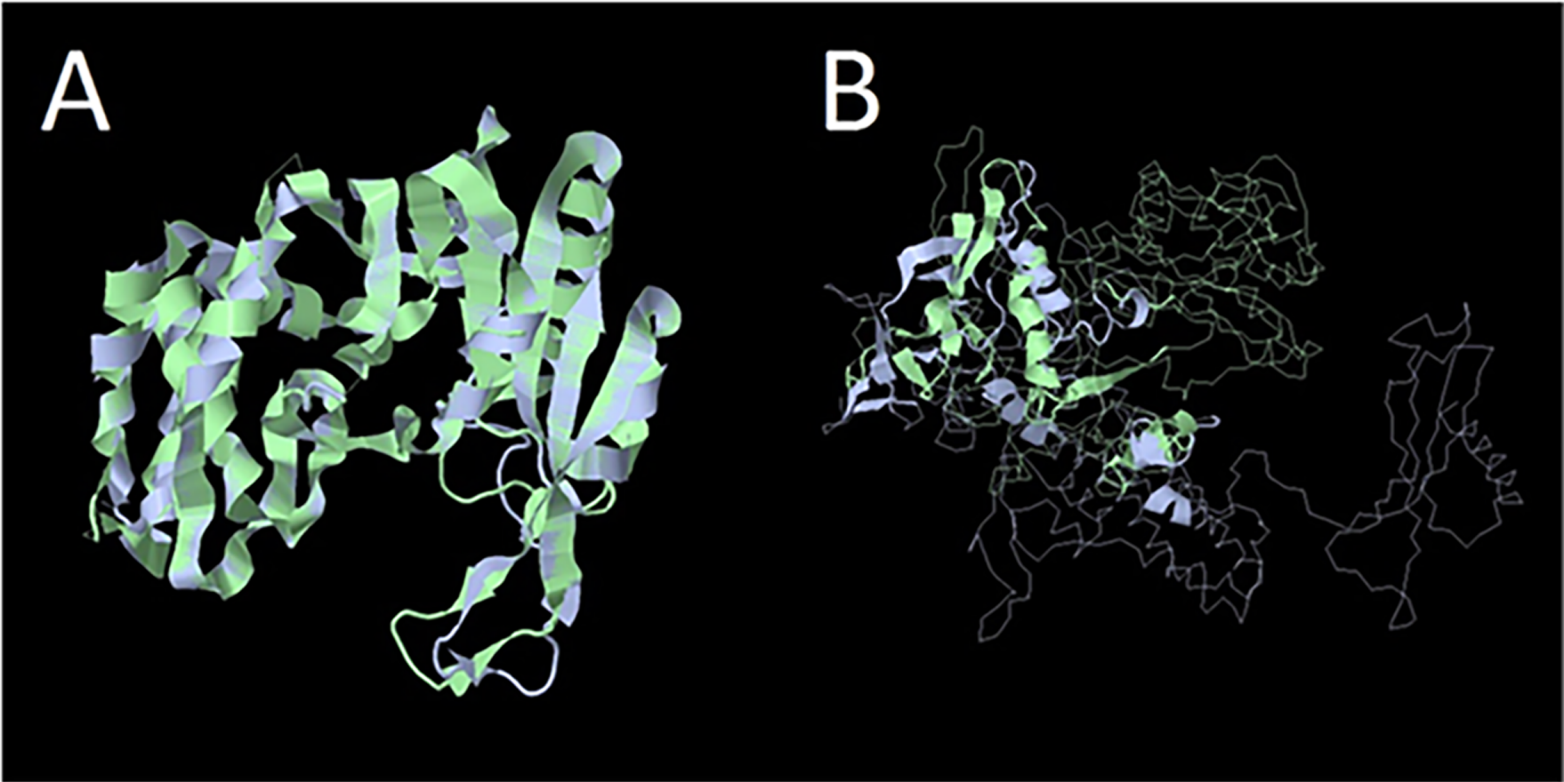
Predicted gp164 RisingSun functional protein modeling using RaptorX [51, 52]. **A)** This model reflects RisingSun gene product 164 aligned to the protein DNA adenine methylase [*Bilophila* sp. 4_1_30]: WP_009733305.1. Both proteins have similar folds, indicating that they may share the same function. **B)** This model reflects RisingSun gene product 85 aligned to the protein RNA polymerase beta subunit [*Erwinia* phage vB_EamM_RAY]: ANH51783.1. The protein folds diverge drastically, indicating that the two proteins may not share a similar function.

Due to the distant nature of many of the BLASTP hits, we analyzed several protein putative functions by comparing their predicted folds to those of their BLASTP hits using RaptorX (Fig 5). Of the 50 RisingSun gene products with a putative function of interest, 26 shared similar folds to at least one of their respective BLASTP hits, suggesting that they share that putative function (S2 Table). Ten of these 26 proteins matched those from bacteria other than *Erwinia* while nine matched those from non-*Erwinia* phages. This leaves over half of these proteins originating from a source other than *Erwinia* or its phages. The remaining 24 proteins do not have enough sequence homology to suggest further conserved function.

### Mass spectrometry

As shown in Table 4, we were able to confirm the accuracy of our genome annotation via mass spectrometry identification of protein fragments within the viral lysate of RisingSun. Mass spectrometry was able to identify nine structural proteins, three proteins with other putative functions, five novel proteins, and 14 hypothetical proteins within the phage lysate. The two proteins that had the most significant coverage and retrieval were gp68 and gp83. The gp83 product is the likely major capsid protein from BLASTP analysis, whereas gp68 has homology (E-value: 3.10e^-07^) to pfam12699 phiKZ-like internal head proteins. Besides phage structural proteins, three proteins with putative function were identified; a putative transglycosylase (gp231) that may aid in breaking down the *Erwini*a cell wall, a putative UvsX-like protein that is likely to function in DNA recombination (gp218), and a tubulin-like protein (gp17). Although a majority (84%) of the peptides identified by mass spectrometry belong to putative hypothetical proteins having homologs in NCBI, five completely novel proteins were identified that have never before been reported (that lack a BLASTP hit with an E-value <10^−7^). These proteins demonstrate the distant nature of this phage when compared to known phages, as 43% of the proteins annotated were novel. Proteins that are under-represented in the mass spectrometry data may indicate either lower expression levels, or proteins whose expression occur in the host but are not present in the virion.

**Table 4:** RisingSun gene products with peptides detected by LC/MS/MS of a crude phage lysate.

RisingSun gp #	Putative Protein Function	Retrieval #*	%Cov/%Cov(95)	Peptides(95%)
**Phage Structural Proteins**				
gp83	major capsid protein	1	75.1/54.88	48
gp68	internal head protein	2	62.65/40.96	80
gp200	virion structural protein	12	36.36/26.22	5
gp204	virion structural protein	13	35.56/25.7	6
gp2	tail tube protein	17	61,41/18.79	4
gp63	phage capsid and scaffold	26	34.33/6.54	2
gp1	tail sheath protein	43	24.12/1.26	1
gp196	virion structural protein	57	52.94/6.23	1
gp66	internal head protein	73	27.61*	0
**Other Putative Functions**				
gp231	transglycosylase	44	53.06/4.76	1
gp218	UsvX recombination protein-like	84	20*	0
gp17	tubulin-like protein	62	41.43/4.36	1
**Novel Hypothetical Proteins (no BLASTP hit E-value of <10**^**−7**^**)**				
gp163		9	64.81/37.96	7
gp206		60	29.38/9.00	1
gp71		54	47.54/3.93	1
gp150		72	11.45*	0
gp72		11	37.77/20.11	10
**Hypothetical Phage Proteins**				
gp28		14	34.19/11.65	5
gp5		15	51.69/13.84	6
gp189		16	40.26/14.29	5
gp59		18	50/8.59	3
gp243		20	46.08/6.14	2
gp185		25	38.59/9.00	2
gp233		27	29.56/4.56	2
gp65		32	18.5/4.62*	1
gp216		33	29.08/1.59*	1
gp73		40	37.26/3.79	1
gp207		51	18.21/5.84	1
gp131	SPFH domain containing protein	55	41.87/5.19	1
gp64		67	43.49/4.75	1
gp212		71	21.25*	0

The gene product number (RisingSun gp #), putative protein function (from BLASTP homolog or conserved domain CD), retrieval number, percent of the protein covered by peptide matches is provided (the percentage is given for both total and 95 percent confidence peptides), and number of 95% peptides is given. *The retrieval numbers missing correspond to bacterial or human (such as keratin) proteins present in the crude lysate. No reverse peptides of RisingSun were detected. The asterisks (*) indicates low confidence proteins due to total coverage of less than 30%.

In work by Lecoutere et. al. the team performed ESI-MS/MS on *Pseudomonas* phages phiKZ and EL [53]. Upon comparing the gene products, they and we have retrieved several different gp have been in common. Of the structural proteins retrieved in RisingSun, gene products 1, 2, 63, 66, and 68 had corresponding proteins retrieved by Lecoutere. Gene products 200 and 204 do not have matches to phage EL according to BLASTP while gp83 and gp196 do have matches but were not retrieved in the other analysis. Gene product 231 with a putative transglycosylase function was also retrieved by both labs while gp17 and 218 were retrieved in our lab. In the hypothetical protein grouping gp131 and 185 did not have matches to phage EL while gp28, 212, and 216 were only retrieved by our group. The rest of the proteins in this group namely gp5, 59, 64, 65, 73, 189, 207, 233, and 243 were obtained by both labs.

#### Motif analysis shows putative structural protein conservation

**Fig 6:**
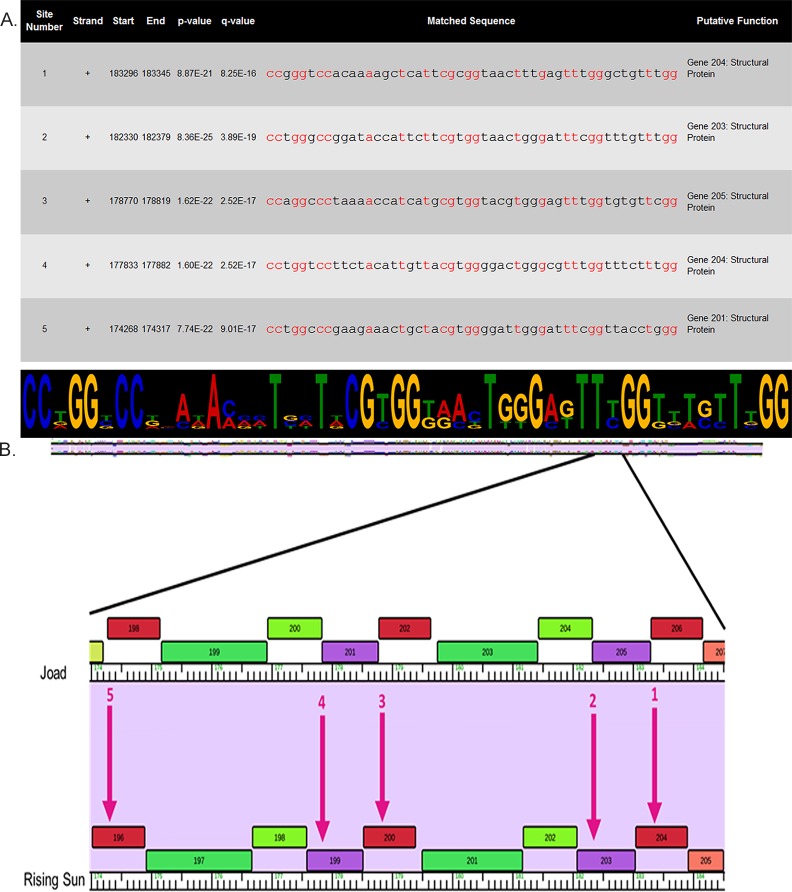
Locations of one motif discovered in five different structural genes. **A)** Exact location of motif sites, p and q-values, sequence of motifs (conserved nucleotides shown in red), and putative gene function. Logogram shows motif sequence and larger letters represent frequent conservation. **B)** Arrows show approximate location of motif site. Genes were analyzed from the RisingSun annotation and thus locations are shown in the RisingSun phamerator map.

Finding conserved motifs in phage genomes may provide insight into transcriptional regulons, and therefore a deeper understanding of phage lifecycle and proteomics due to the frequent co-regulation of related genes [54]. RisingSun was analyzed for motifs using The Center for Phage Technology Galaxy Server [55], a software that allows you to search an entire genome at once. After covering the entire genome only one motif passed our e-value significance threshold of 10^−7^. We then ran this motif through FIMO [40], a program that allows the search of an entire genome for a specific motif. We looked for occurrences of the motif represented by a logogram in (Fig 6A), which passed a q-value significance threshold of 0.01. Only 5 repetitions of this motif occurred and all in a relatively small area near the end of the genome (Fig 6B).

Identifying genes neighboring these motifs may identify co-regulated genes with related function. Fig 6. is a portion of the Joad and RisingSun genome map showing the motif locations within a span of 10 genes. Five gene products, namely gp204, gp203, gp200, gp199 and gp196 contain the motifs suggesting gene products 196–205 may be co-regulated. One of the gene products (gp205) in this region is completely novel (no BLASTP hit), and nine (gp204, gp203, gp202, gp201, gp200, gp199, gp198, gp197 and gp196) are putative virion/structural proteins based on annotation of related phage BLASTP hits. Despite the novelty of one of the genes, the evidence that this conserved motif brings suggests that genes gp196-205 are indeed structural proteins or are involved in virion assembly and that all are transcribed together. These results, however, are hypothetical and wet lab experiments are necessary for confirmation.

## Conclusions

Herein we report the discovery and characterization of two newly isolated *Erwinia* phages, Joad and RisingSun, bringing the apparent total number of *Erwini*a phages on NCBI as of writing of this article to 45 reported full genome sequences. Joad and RisingSun, at over 200kbp, are two highly related Jumbo phages of the *Myoviridae* family, only distantly related to *Pseudomonas* phages EL and OBP and even more distantly related to *Vibrio* phages VP4B and pTD1 (Fig 2A). All six of these phages are most likely distant phiKZ-like phages as previously determined for EL and OBP [56, 57]. Dot Plot, ANI and BLASTP analysis all suggest the unique nature of Joad and RisingSun phages but also brings to attention that these phages have closest similarity to phages that infect bacteria outside of the *Enterobacteriales* order of bacteria. A BLASTP analysis of the putative proteome reveals BLASTP hits for ~57% of the proteins, however 33% are proteins of unknown function. The remaining putative gene products with no BLASTP hits (43%) represent proteins that have not been previously found in nature. These join the bounty of phage gene products [49, 50] with unknown structural folds and function.

Both *Pseudomonas* and *Vibrio* phages had higher similarity to Joad and RisingSun than any of the >800 *Enterobacteriaceae* phages on NCBI, despite all three of their respective hosts phylogenetically only having the class *Gammaproteobacteria* in common [58]. Of the 140 gene products with a BLASTP hit, 81% and 63% of the closest hits corresponded to gene products from *Pseudomonas* and *Vibrio* phages, respectively. Given that *Pseudomonas* bacteria are found on apple blossoms [59–64] it is reasonable to posit that extensive exposure to this strain has allowed for lateral gene transfer between phages, with possible tail fiber exchange for host recognition [65–69]. However, recent work by Adeolu and colleagues has indicated that the *Enterobacteriales* order is more nuanced than previously thought and that classification methods may need to be rethought [10]. In addition to a standard 16S rRNA based phylogenetic tree for the order *Enterobacteriales*, Adeolu and colleagues constructed three more trees based on 1548 core proteins, 53 ribosomal proteins, or four multi-locus sequence analysis proteins. After analyzing 179 species within this order they propose forming seven families, one of which is an *Erwinia-Pantoea* clade [10]. This classification is supported by our findings that these phages are quite distinct from other Enterobacteriaceae phages and can infect a *Pantoea vegans* strain. A reclassification of this magnitude highlights the complex evolution, and hence classification, of bacteria and the phage that infect them. Because phages are a major source of bacterial evolution, through the control of bacterial number as well as lateral gene transfer, understanding their complexity is vital. Joad and RisingSun join the 43 other *Erwinia* phages available on NCBI and provide insight into this evolutionary complexity, highlighting the similarity between *Erwinia* and the *Pseudomonads*. The relationship of these phages with more distant bacteria may reflect both an ecological niche as well as true diversity within the *Enterobacteriales*.

## Supporting information

S1 Table A list of RisingSun (MF459646) gene products that are in common with Pseudomonas phages EL (NC_007623) and OBP (NC_016571) as well as Vibrio phages pTD1 (AP017972) and VP4B(KC131130). BLASTP was used to determine the gene products in common, using the e-value cutoff of 1e-7. Putative functions and the gene product number are provided from the annotation of phage RisingSun (from both BLASTP hits and conserved domains retrieved). The symbol “x” indicates no homolog was found. Bolded gp# indicates that the putative function was taken from the phage of that column.
(DOCX)

S2 Table Predicted protein fold of putative RisingSun functional proteins as compared to BLASTP functional hits. Putative gene products identified for RisingSun were compared to their respective BLASTP hit with known function using RaptorX. Each alignment was given an average template modeling score (TM) between 0 and 1. According to Sheng Wang et al., if the TM is greater than 0.6, there is a 90% chance that two proteins share a similar fold. However, if the TM is less than 0.4, then the two proteins are not similar. When considering 50 genes with a putative protein function of interest, 26 proteins demonstrated similar folding to at least one of their respective BLASTP hit. Three proteins demonstrated similar folding to two BLASTP hits, for a given pair the structures were compared using RaptorX. In each of the three cases the protein structures matched each other having TM values above 0.6.
(DOCX)
