# Supplementary material for: Characterization of two related Erwinia myoviruses that are distant relatives of the PhiKZ-like Jumbo phages
Source: PLoS One. 2018 Jul 6;13(7):e0200202. doi: 10.1371/journal.pone.0200202 (PMC6034870; doi:10.1371/journal.pone.0200202)
Supplement: S1 Table — BLASTP was used to determine the gene products in common, using the e-value cutoff of 1e-7. Putative functions and the gene product number are provided from the annotation of phage RisingSun (from both BLASTP hits and conserved domains retrieved). The symbol “x” indicates no homolog was found. Bolded gp# indicates that the putative function was taken from the phage of that column. (DOCX) [file pone.0200202.s001.docx]

**Supplementary Table 1. A list of RisingSun (MF459646) gene products that are in common with Pseudomonas phages EL (NC_007623) and OBP (NC_016571) as well as Vibrio phages pTD1 (AP017972) and VP4B(KC131130).**

| **Gene products common with EL** | **Gene products in common with OBP** | **Gene products in common with pTD1** | **Gene products in common with VP4B** | **Putative function** |
| --- | --- | --- | --- | --- |
| gp1 | gp1 | gp1 | gp1 | Tail sheath protein |
| gp2 | gp2 | gp2 | gp2 | Tail tube protein |
| gp3 | gp3 | gp3 | gp3 | NKF |
| gp4 | gp4 | gp4 | gp4 | Structural head protein |
| gp5 | x | x | x | NKF |
| x | x | gp6 | x | dTMP kinase |
| gp17 | gp17 | x | x | Tubulin-like protein |
| gp24 | gp24 | gp24 | gp24 | NKF |
| gp25 | gp25 | gp25 | gp25 | T4-like DNA polymerase |
| gp27 | gp27 | gp27 | gp27 | NKF |
| gp28 | gp28 | gp28 | gp28 | NKF |
| gp29 | gp29 | gp29 | gp29 | RNA polymerase β' subunit |
| gp30 | gp30 | gp30 | gp30 | NKF |
| gp34 | x | gp34 | gp34 | NKF |
| gp35 | gp35 | gp35 | gp35 | NKF |
| gp36 | gp36 | gp36 | gp36 | Nuclease SbcCD, D subunit |
| gp37 | gp37 | gp37 | gp37 | NKF |
| gp38 | gp38 | gp38 | gp38 | NKF |
| gp39 | gp39 | gp39 | gp39 | NKF |
| gp40 | x | gp40 | gp40 | NKF |
| gp41 | gp41 | gp41 | gp41 | NKF |
| x | gp42 | gp42 | gp42 | NKF |
| gp43 | gp43 | gp43 | gp43 | DNA directed RNA polymerase ß subunit |
| gp44 | gp44 | gp44 | ***gp44*** | DNA-directed RNA polymerase subunit ß '- ß' |
| gp45 | gp45 | ***gp45*** | gp45 | Phage terminase, large subunit |
| gp50 | gp50 | gp50 | gp50 | DNA directed RNA polymerase ß |
| gp51 | gp51 | gp51 | gp51 | NKF |
| gp52 | x | x | x | NKF |
| gp53 | gp53 | gp53 | gp53 | ZipA-like (cell division protein) |
| gp54 | x | x | x | NKF |
| gp55 | x | x | x | NKF |
| gp56 | gp56 | gp56 | gp56 | DNA polymerase |
| gp57 | gp57 | gp57 | gp57 | NKF |
| gp58 | x | x | x | NKF |
| gp59 | x | x | x | NKF |
| gp61 | gp61 | gp61 | gp61 | Virion structural protein |
| gp62 | gp62 | gp62 | gp62 | Structural protein |
| gp63 | gp63 | ***gp63*** | gp63 | Phage capsid and scaffold |
| gp64 | gp64 | x | gp64 | NKF |
| gp65 | x | gp65 | gp65 | NKF |
| gp66 | x | gp66 | gp66 | phiKZ-like internal head protein |
| gp67 | x | gp67 | gp67 | NKF |
| gp68 | gp68 | gp68 | gp68 | phiKZ-like internal head protein |
| gp73 | x | x | x | NKF |
| gp75 | gp75 | gp75 | gp75 | NKF |
| gp76 | gp76 | gp76 | gp76 | Virion structural protein |
| gp77 | gp77 | gp77 | gp77 | NKF |
| gp78 | gp78 | gp78 | gp78 | Virion structural protein |
| gp81 | gp81 | ***gp81*** | gp81 | Phage DNA helicase |
| gp82 | x | x | x | NKF |
| gp83 | gp83 | gp83 | gp83 | Phage capsid and scaffold |
| gp84 | x | x | x | NKF |
| gp85 | gp85 | gp85 | gp85 | RNA polymerase β subunit |
| gp86 | x | x | x | NKF |
| gp88 | gp88 | gp88 | gp88 | Virion structural protein |
| gp89 | gp89 | gp89 | gp89 | Virion structural protein |
| gp91 | gp91 | gp91 | gp91 | Virion structural protein |
| gp93 | gp93 | gp93 | gp93 | Holliday junction resolvase |
| gp94 | x | gp94 | gp94 | NKF |
| x | gp95 | x | x | NKF |
| gp97 | gp97 | gp97 | gp97 | Virion structural protein |
| gp98 | gp98 | ***gp98*** | gp98 | Phage tail fiber protein |
| gp99 | gp99 | gp99 | gp99 | Virion structural protein |
| gp100 | gp100 | gp100 | gp100 | Virion structural protein |
| gp101 | x | x | x | NKF |
| gp102 | gp102 | gp102 | gp102 | Structural protein |
| gp103 | gp103 | ***gp103*** | gp103 | DNA double strand break repair |
| x | gp104 | x | x | Lytic transglycosylase |
| ***gp108*** | x | gp108 | gp108 | NAD-dependent DNA ligase |
| gp112 | x | gp112 | x | Ribonuclease HI |
| gp120 | x | gp120 | gp120 | Structural protein |
| x | gp147 | x | x | Serine/Threonine protein phosphatase |
| gp166 | gp166 | x | x | NKF |
| x | x | gp180 | gp180 | Helix turn helix XRE-family domain |
| gp189 | x | x | x | NKF |
| ***gp190*** | gp190 | gp190 | x | GroEL |
| x | x | gp192 | ***gp192*** | Thymidine kinase |
| gp197 | x | x | x | Virion structural protein |
| gp201 | x | x | x | Virion structural protein |
| gp205 | x | x | x | NKF |
| gp207 | x | x | x | NKF |
| gp211 | gp211 | ***gp211*** | gp211 | Phage DNA helicase |
| gp212 | x | x | x | NKF |
| gp213 | gp213 | gp213 | gp213 | NKF |
| gp214 | gp214 | gp214 | gp214 | Virion structural protein |
| gp215 | x | x | x | NKF |
| gp216 | gp216 | gp216 | gp216 | NKF |
| gp217 | x | gp217 | gp217 | NKF |
| gp218 | gp218 | gp218 | gp218 | UvsX protein |
| gp219 | x | gp219 | gp219 | NKF |
| gp220 | gp220 | gp220 | gp220 | Virion Structural protein |
| gp222 | x | gp222 | x | NKF |
| gp223 | gp223 | gp223 | gp223 | NKF |
| gp225 | gp225 | x | gp225 | NKF |
| gp226 | gp226 | gp226 | gp226 | NKF |
| gp227 | gp227 | gp227 | gp227 | Structural protein |
| gp228 | gp228 | ***gp228*** | gp228 | Chitinase and glycoside hydrolase |
| gp229 | gp229 | gp229 | gp229 | DNA directed RNA polymerase ß subunit |
| gp230 | gp230 | gp230 | gp230 | RNA polymerase β subunit |
| gp231 | x | gp231 | gp231 | D-alanyl-D-alanine carboxypeptidase |
| gp232 | x | x | x | NKF |
| gp233 | x | gp233 | x | NKF |
| gp234 | gp234 | gp234 | gp234 | NKF |
| gp235 | gp235 | gp235 | gp235 | Virion structural protein |
| gp236 | x | x | x | NKF |
| gp237 | x | gp237 | gp237 | NKF |
| x | gp238 | gp238 | gp238 | Phosphohydrolase |
| ***gp240*** | gp240 | gp240 | gp240 | ATP-dependent DNA helicase |
| gp241 | gp241 | gp241 | gp241 | Virion Structural protein |
| gp242 | gp242 | gp242 | gp242 | Virion Structural protein |
| gp243 | gp243 | gp243 | gp243 | NKF |

BLASTP was used to determine the gene products in common, using the e-value cutoff of 1e-7. Putative functions and the gene product number are provided from the annotation of phage RisingSun (from both BLASTP hits and conserved domains retrieved). The symbol “x” indicates no homolog was found. Bolded gp# indicates that the putative function was taken from the phage of that column.
