# Supplementary material for: Characterization of two related Erwinia myoviruses that are distant relatives of the PhiKZ-like Jumbo phages
Source: PLoS One. 2018 Jul 6;13(7):e0200202. doi: 10.1371/journal.pone.0200202 (PMC6034870; doi:10.1371/journal.pone.0200202)
Supplement: S2 Table — Putative gene products identified for RisingSun were compared to their respective BLASTP hit with known function using RaptorX. Each alignment was given an average template modeling score (TM) between 0 and 1. According to Sheng Wang et al., if the TM is greater than 0.6, there is a 90% chance that two proteins share a similar fold. However, if the TM is less than 0.4, then the two proteins are not similar. When considering 50 genes with a putative protein function of interest, 26 proteins demonstrated similar folding to at least one of their respective BLASTP hit. Three proteins demonstrated similar folding to two BLASTP hits, for a given pair the structures were compared using RaptorX. In each of the three cases the protein structures matched each other having TM values above 0.6. (DOCX) [file pone.0200202.s002.docx]

**Supplementary Table 2**. **Predicted protein fold of putative RisingSun functional proteins as compared to BLASTP functional hits.**

| **GP#** | **Hypothetical function** | **Phage alignment** | **Fold** | **TM** |
| --- | --- | --- | --- | --- |
| 1 | Tail sheath protein | *Erwinia* phage vB_EamM_Simmy50 | N | 0.40 |
| 2 | Tail tube protein | *Erwinia* phage Ea35-70 | N | 0.18 |
| 6 | dTMP kinase | *Candidatus Blackburnbacteria* | Y | 0.84 |
| 7 | Thymidylate kinase | *Escherichia coli* | Y | 0.90 |
| 29 | RNA pol β’ subunit | *Erwinia* phage vB_EamM_Simmy50 | Y | 0.80 |
| 36 | Nuclease SbcCD, D subunit | *Erwinia* phage vB_EamM_Deimos-Minion | Y | 0.81 |
| 43 | DNA-directed RNA pol β subunit | *Erwinia* phage vB_EamM_Special G | Y | 0.74 |
| 44 | DNA-directed RNA pol subunit β’- β” | *Vibrio* phage VP4B | Y | 0.60 |
| 44 | Phage terminase, large subunit | *Vibrio* phage pTD1 | Y | 0.60 |
| 44 | ATP-dependent DNA helicase | *Pseudomonas* phage EL | N | 0.24 |
| 45 | Phage terminase, large subunit | *Vibrio* phage pTD1 | N | 0.23 |
| 50 | DNA-directed RNA pol β | *Erwinia* phage vB_EamM_Deimos-Minion | Y | 0.64 |
| 53 | Cell division protein ZipA | *Escherichia coli* | N | 0.26 |
| 56 | DNA polymerase | *Erwinia* phage vB_EamM_Simmy50 | Y | 0.79 |
| 63 | Phage capsid and scaffold | *Vibrio* phage pTD1 | N | 0.38 |
| 66 | phiKZ-like phage internal head protein | *Pseudomonas* phage phiKZ | N | 0.24 |
| 68 | phiKZ-like phage internal head protein | *Pseudomonas* phage phiKZ | N | 0.23 |
| 81 | Helicase | *Vibrio* phage pTD1 | Y | 0.87 |
| 83 | Major capsid protein | *Vibrio* phage pTD1 | N | 0.12 |
| 85 | RNA pol β subunit | *Erwinia* phage vB_EamM_RAY | N | 0.12 |
| 93 | Holliday junction resolvase | *Pseudomonas* phage Phabio | Y | 0.87 |
| 98 | Tail fiber protein | *Vibrio* phage pTD1 | N | 0.19 |
| 103 | Double strand break repair | *Vibrio* phage pTD1 | N | 0.34 |
| 104 | Lytic transglycosylase | *Erwinia* phage PhiEaH1 | Y | 0.86 |
| 104 | Endolysin | *Erwinia* phage vB_EamM_EarlPhillipIV | Y | 0.84 |
| 108 | NAD-dependent DNA ligase | *Pseudomonas* phage EL | Y | 0.86 |
| 112 | Ribonuclease HI | *Pseudomonas aeruginosa* | Y | 0.95 |
| 119 | Type 3 dihydrofolate reductase | *Vibrio litoralis* | Y | 0.93 |
| 124 | Phage exonuclease | *Vibrio splendidus* | Y | 0.78 |
| 131 | SPFH domain/band 7 family protein | *Pseudomonas* phage vB_PaeM_PS24 | N | 0.52 |
| 136 | SGNH/GDSL hydrolase family protein | *Salmonella enterica* | N | 0.53 |
| 136 | Head outer capsid protein | *Enterobacter* phage CC31 | N | 0.50 |
| 139 | Phosphate starvation protein PhoH | *Citrobacter amalonaticus* | Y | 0.78 |
| 139 | ATPase | *Buttiauxella noackiae* | Y | 0.90 |
| 147 | Serine/threonine protein phosphatase | *Yersinia* phage phiR201 | Y | 0.92 |
| 153 | Membrane protein | *Pantoea dispersa* | N | 0.18 |
| 160 | Nucleoside triphosphate pyrophosphohydrolase | *Staphylococcus aureus* | Y | 0.92 |
| 164 | DNA adenine methylase | *Bilophila* | Y | 0.98 |
| 180 | Helix turn helix XRE-family domain | *Brachyspira intermedia* | N | 0.29 |
| 186 | Thymidylate synthase | *Erwinia* phage vB_EamM_ChrisDB | N | 0.57 |
| 187 | Homing endonuclease GIY-YIG family | *Acinetobacter* phage Ac42 | N | 0.33 |
| 188 | Thymidylate synthase | *Cronobacter sakazakii* | Y | 0.97 |
| 190 | GroEL-like chaperonine protein | *Pseudomonas* phage EL | N | 0.55 |
| 192 | Thymidine kinase | *Vibrio* phage VP4B | Y | 0.86 |
| 193 | Carbohydrate-binding protein | *Brenneria* sp. EniD312 | N | 0.42 |
| 193 | Coagulation factor 5/8 type domain protein | *Pectobacterium carotovorum* | N | 0.43 |
| 194 | Tail-fiber protein | *Pectobacterium* phage ZF40 | N | 0.15 |
| 195 | Carbohydrate-binding protein | *Brenneria* sp. EniD312 | N | 0.50 |
| 195 | Coagulation factor 5/8 type domain protein | *Pectobacterium carotovorum* | N | 0.43 |
| 211 | Phage associated helicase | *Vibrio* phage pTD1 | N | 0.32 |
| 218 | UvsX protein | *Pseudomonas* phage PhiPA3 | Y | 0.71 |
| 220 | RNA pol β subunit | *Ralstonia* phage RSF1 | Y | 0.74 |
| 229 | DNA-direct RNA pol β subunit 2 | *Erwinia* phage vB_EamM_Special G | N | 0.51 |
| 230 | RNA pol β subunit | *Erwinia* phage vB_EamM_Special G | N | 0.37 |
| 231 | D-alanyl-D-alanine carboxypeptidase | *Erwinia* phage vB_EamM_Phobos | N | 0.46 |
| 231 | Transglycosylase | *Erwinia* phage PhiEaH1 | Y | 0.80 |
| 238 | Phosphohydrolase | *Erwinia* phage vB_EamM_Simmy50 | Y | 0.79 |
| 240 | Putative ATP-dependent DNA helicase | *Pseudomonas* phage EL | N | 0.43 |
| 240 | Terminase, large subunit | *Erwinia phage EA35-70* | Y | 0.61 |

Putative gene products identified for RisingSun were compared to their respective BLASTP hit with known function using RaptorX. Each alignment was given an average template modeling score (TM) between 0 and 1. According to Sheng Wang et al., if the TM is greater than 0.6, there is a 90% chance that two proteins share a similar fold. However, if the TM is less than 0.4, then the two proteins are not similar. When considering 50 genes with a putative protein function of interest, 26 proteins demonstrated similar folding to at least one of their respective BLASTP hit. Three proteins demonstrated similar folding to two BLASTP hits, for a given pair the structures were compared using RaptorX. In each of the three cases the protein structures matched each other having TM values above 0.6.
